# FPGA-Based HD Camera System for the Micropositioning of Biomedical Micro-Objects Using a Contactless Micro-Conveyor

**DOI:** 10.3390/mi8030074

**Published:** 2017-03-02

**Authors:** Elmar Yusifli, Reda Yahiaoui, Saeed Mian Qaisar, Mahmoud Addouche, Basil Al-Mahdawi, Hicham Bourouina, Guillaume Herlem, Tijani Gharbi

**Affiliations:** 1 Nanomedicine Lab, University de Bourgogne Franche-Comte, Besancon 25000, France; basil.al-mahdawi@univ-fcomte.fr (B.A.-M.); guillaume.herlem@univ-fcomte.fr (G.H.); tijani.gharbi@univ-fcomte.fr (T.G.); 2 Institut Femto-ST, University de Bourgogne Franche-Comte, Besancon 25000, France; mahmoud.addouche@femto-st.fr; 3 Electrical and Computer Engineering Department, Effat University, Jeddah 21478, Saudi Arabia; 4 Physics Laboratory, Ecole Normale Supérieure-Bou Saada, 28200 M’Sila, Algeria; hi.bourouina@gmail.com

**Keywords:** Field Programmable Gate Array (FPGA), VHSIC Hardware Description Language (VHDL), camera driver, embedded design, micro-conveyor, image processing, C++ programming

## Abstract

With recent advancements, micro-object contactless conveyers are becoming an essential part of the biomedical sector. They help avoid any infection and damage that can occur due to external contact. In this context, a smart micro-conveyor is devised. It is a Field Programmable Gate Array (FPGA)-based system that employs a smart surface for conveyance along with an OmniVision complementary metal-oxide-semiconductor (CMOS) HD camera for micro-object position detection and tracking. A specific FPGA-based hardware design and VHSIC (Very High Speed Integrated Circuit) Hardware Description Language (VHDL) implementation are realized. It is done without employing any Nios processor or System on a Programmable Chip (SOPC) builder based Central Processing Unit (CPU) core. It keeps the system efficient in terms of resource utilization and power consumption. The micro-object positioning status is captured with an embedded FPGA-based camera driver and it is communicated to the Image Processing, Decision Making and Command (IPDC) module. The IPDC is programmed in C++ and can run on a Personal Computer (PC) or on any appropriate embedded system. The IPDC decisions are sent back to the FPGA, which pilots the smart surface accordingly. In this way, an automated closed-loop system is employed to convey the micro-object towards a desired location. The devised system architecture and implementation principle is described. Its functionality is also verified. Results have confirmed the proper functionality of the developed system, along with its outperformance compared to other solutions.

## 1. Introduction

Micro-object conveyance is becoming an essential part of different key sectors, including biomedical, nuclear medicine, multimedia, IT equipment, etc. In this context, much interesting research is in progress. Positioning and the micromanipulation of small objects are essential in micro manufacturing and micro-robotics sectors. Thus, various conveyance techniques have been presented [1,2,3,4,5,6,7].

Classic conveyance techniques require contact with the object. During manipulation, this can raise problems including object contamination and deterioration [8]. Medicinal production has very detailed guidelines to lower the possibility of product contamination via external contact. These recommendations are very restrictive and need complex and costly solutions, especially in the case of systems that employ conveyance by contact [5,6,9,10,11,12,13,14,15]. A cost effective and reliable solution can be developed by employing contactless conveyors.

Similarly, in IT equipment, nuclear medicine and multimedia manufacturing sectors, various fragile micro-objects are employed [16]. Conveyance with contact can easily cause object deterioration. Therefore, contactless conveyors are also good candidates for this sector.

Contactless conveyors mostly work on the principle of airflow levitation, which avoids contact with conveyed objects. Therefore, it tends to solve contamination and deterioration problems occurring due to conveyance by contact [1,2,3,4,5,6,7]. This keeps the system efficient in terms of cost and complexity. Along with these interesting characteristics, contactless conveyors should also keep the interesting features of conveyors with contact, such as conveyance speed, precision, etc. [17].

To implement these conveyance techniques, different types of Microelectromechanical Systems (MEMS) based actuator arrays have been developed. Certain proposed systems are based on pneumatic [2,3,4] and magnetic [5] techniques. Others are based on the thermal-bimorph and electrostatic [6] techniques. In [1], a micro-conveyor concept and its implementation is proposed. This system is useful for the manipulation of small planar objects. This micro-conveyor is briefly described in Section 2.1. The proposed micro-conveyor has several advantages over conventional ones. It employs an array of air jets to handle small objects and therefore has the advantage of not requiring precision gripping or particular contact configuration, which may be difficult to realize for small objects. Moreover, during manipulation, only a minor friction force occurs, thanks to the aerodynamic levitation of the object, which results in faster object manipulation.

In [7], a sensor less manipulation scheme is discussed. It is based on the Goldberg’s algorithm for parallel jaw grippers. In [8], Bohringer has extended this work by opposing directions of MEMS ciliary actuators. However, the system still lacks any feedback and command [8]. The absence of feedback and command can lead to uncertain behaviors [9]. Moreover, the MEMS actuator arrays have to be programmed every time as a function of the targeted object features. In addition, this kind of control is only applicable to ciliary actuators and cannot be used with pneumatic ones [10].

Recent works focus on improving the system performance by including feedback and automated control and command, which requires including sensors, for feedback, and automated control and command along with the array of actuators in the system. Based on this principle, Biegelsen [18] has developed an array of sensors and actuators that can manipulate paper sheets using air jets. In [19], Laurent et al. have presented an automatic control system for the “smart surface” [1]. The proposed system is based on a standard USB camera and a MATLAB-based application for automatic control, which makes their system MATLAB license dependent and Personal Computer (PC) based. Dang [20] has proposed contactless micro-conveyer systems. These solutions employ a commercial camera as a sensor. It tracks the object position and transmits data to the image processing and control module, and it runs on a PC. The image processing and control module makes decisions and generates commands accordingly to pilot the employed array of actuators. A series of actuations are performed by the actuator to move the object towards the desired location. Zawadzki [21] used a FPGA-based system for the real-time tracking of a moving object. Ataka [9] has used a charge coupled device (CCD) camera and a two-dimensional conveyance system to convey a micro-object. They employ a contact-based conveyer for the object manipulation. Perez et al. [22] used a CCD camera to control robot arm with an FPGA-based front-end controller. This is an automated system to control the object by robot arm and therefore belongs to the class of contact-based conveyors.

With the recent technological advancements, the size, cost and power consumption of image sensors, MEMS actuators and FPGAs is constantly decreasing. Therefore, it becomes highly feasible to implement a FPGA-based smart conver, employing a compact camera as a sensor along with the array of MEMS actuators for the manipulation of objects. Inspired by these recent advancements, the proposed micro-object conveyer consists of a camera-based sensor, smart surface-based actuators [1], a FPGA-based embedded front-end processor and an Image Processing, Decision Making and Command (IPDC) module. The front-end processor employs sensors data and pilots actuators to move each object towards the desired location by following the IPDC commands. The actuators are macro-scale and have fixed locations [1,19]. They generate airflow in only one direction [1]. Therefore, four actuators are grouped to blow in a controlled sequence so the targeted object can be moved in two dimensions of freedom [1,19]. The actuators cover a surface of 6.45 cm^2^. The system can convey micro-objects with maximum dimensions of 5 mm × 5 mm and maximum weight of 5 g. A further description of the proposed system is given in Section 2.

## 2. The Proposed System

**Figure 1:**
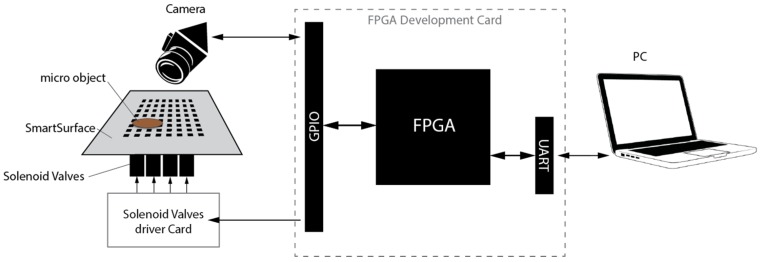
The system block diagram.

The devised micro conveyor system block diagram is shown in Figure 1. A description of the different system modules is provided in the following sub sections.

Figure 1 shows that the proposed system employs the smart surface as an array of actuators [1] and the OmniVision HD camera as a sensor, for real-time object position detection. The sensor output is transmitted to the IPDC module via the FPGA-based front-end processor. The IPDC module compares this data with the operator instruction. Based on this comparison, commands are generated by the IPDC. These commands are transmitted to the front-end processor, which pilots a series of actuation patterns necessary for the placement of the targeted object on the desired location. A specific hardware design and development makes the system efficient in terms of cost and power consumption. Moreover, the re-programmability of FPGAs keeps the system configurable and it can be easily optimized for targeted application.

### 2.1. The Actuator

**Figure 2:**
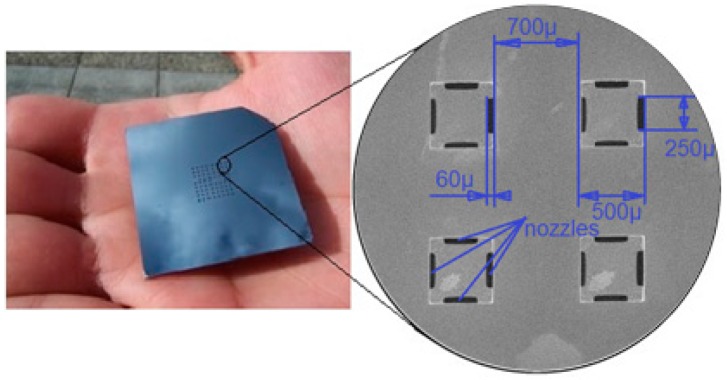
Top side of the titled air jet array.

The array of actuators consists of a stack of three layers, two silicon wafers bonded together by the gold eutectic bonding method and an anodically bonded lower Pyrex glass wafer [1]. Both sides of the silicon wafers are micro-machined using Deep Reactive Ion Etching (DRIE). The top side of the upper silicon wafer represents the manipulation surface and includes an array of 8 × 8 square holes. The process is clear from Figure 2.

**Figure 3:**
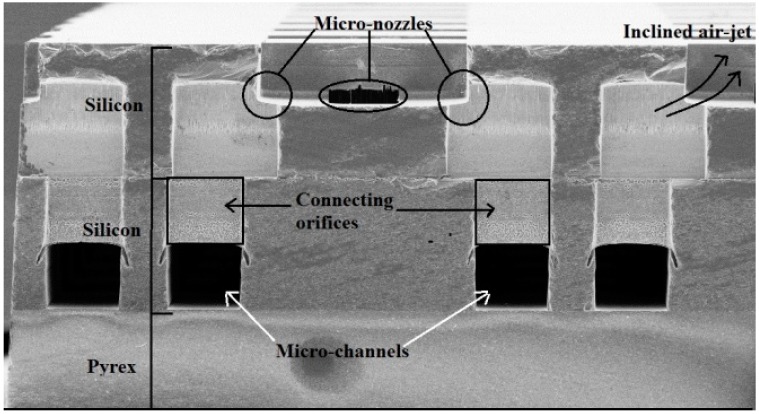
Cross-section view of the wafers (East–West nozzles).

Its bottom side includes 16 microchannels and a set of 8 × 16 rectangular holes. The top side of the lower silicon wafer includes 8 × 16 rectangular holes in similar locations, which are linked to 16 microchannels etched into its bottom side (cf. Figure 3). The wafer also comprises of two holes in order to link the upper wafer channels with the Pyrex layer. Furthermore, common air reservoirs are designed. This is done in order to achieve a homogeneous distribution of the air flow into the channels at the head of each micro-channel [1].

**Figure 4:**
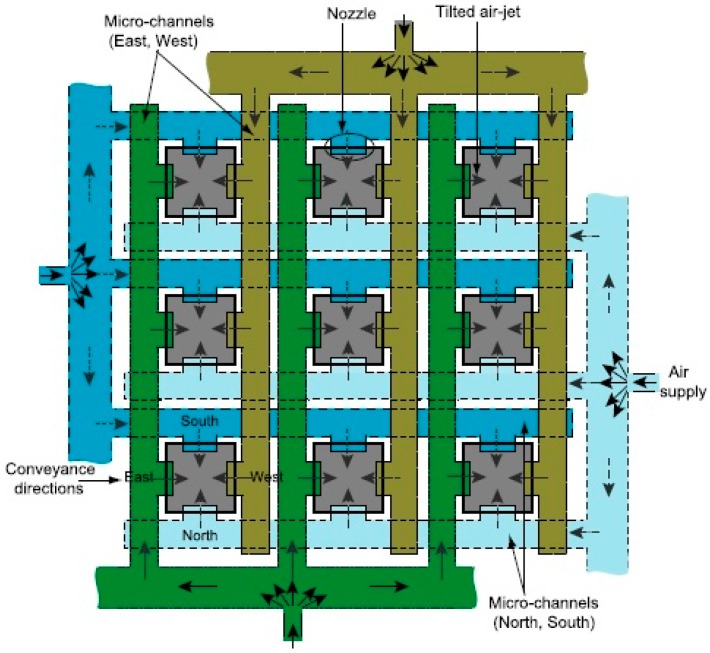
The system network of microchannels.

The Pyrex wafer is used to hermetically seal the microchannels of the lower silicon wafer. Four holes are machined into the Pyrex wafer by ultrasonic machining. This is done in order to connect the system to the air source by means of commercial micro-fluidic connectors, glued onto the back-side of the glass wafer. The system network of microchannels [23,24] is shown in Figure 4.

The array of actuators includes a set of micro-conveyors where each micro-conveyor is composed of a square orifice and four nozzles. The air-flow comes from the back-side through an array of microchannels connected to the micro-nozzles. The compressed air flows throughout common channels, then the air is distributed in the micro-channel and is ejected by the nozzles. The nozzles corresponding to a conveyance way are linked together and blow simultaneously in the same direction. The air jets’ orientation is due to the shift of the holes in the upper wafer (cf. Figure 3). The specific form of these slots permits the air to blow with an angle between 20° and 90° [1].

**Figure 5:**
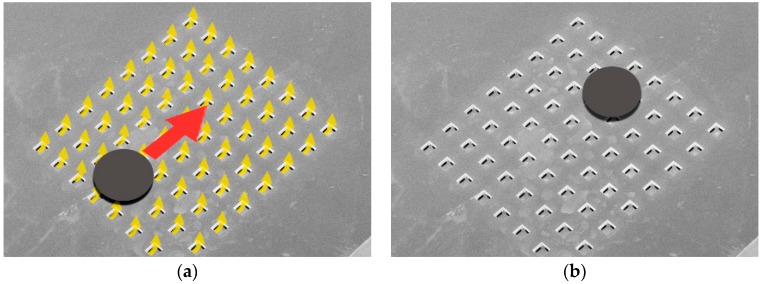
Working principle of the tilted-air-jet array. (**a**) Blowing to one direction. (**b**) Movement of the object.

This design allows lifting and moving the millimeter-sized planar objects in the four cardinal directions. Figure 5 shows the working principle of the tilted-air-jet array. An object of appropriate weight and dimension (cf. Section 1) can be conveyed to a desired position with intermittent air flow pulses of a pre-adjustable pressure up to 20 kPa [1].

In [1], the focus is on the microfabrication of the MEMS (smart surface) device. This device is able to move small objects in four directions without any external contact. The proposed device is already tested with basic, manual and non-automatized systems [19]. In this paper, a fully, FPGA-based, embedded processor is devised for systematically piloting the smart surface in order to maneuver the micro-objects. It is a closed-loop system. The feedback, regarding the micro-object’s current location, is provided via the embedded camera to the IPDC module. The IPDC module compares this micro-object location with the desired one and generates actuation commands. Following these commands, the front-end, FPGA-based, embedded processor generates actuation patterns for the smart surface. The same process is repeated in a closed-loop fashion, unless the micro-object achieves the desired position.

### 2.2. The Sensor Module

The OV*9655* CMOS 1.3Mpx HD camera (OmniVision, Sunnyvale, CA, USA) is employed as an image sensor (cf. Figure 1). It has machine pilotable configuration control such as gain control (GC), white balance (WB), band filter (BF), black level calibration (BLC), color saturation, hue, gamma, sharpness controls, anti-blooming, frame synchronization etc. Its image resolution and frame rate can also be configured according to the targeted application requirements. There is a tradeoff between the camera resolution and the achievable frame rate. The maximum image resolution is 1280 × 1024 pixels. For such a resolution, it can capture 30 fps (Frames per Second). However, a higher frame rate can be achieved for lower resolution modes [25].

### 2.3. The Front End Embedded System (FEES)

**Figure 6:**
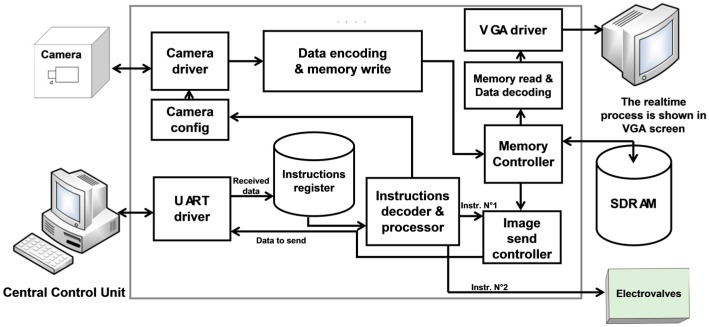
The devised Front End Embedded System (FEES) architecture.

The devised Front End Embedded System (FEES) block diagram is shown in Figure 6. It is implemented on the EP2C20F484 FPGA module, mounted on the DE1 board [26,27]. The FPGA choice is made according to the system requirements such as operating frequency, logic resources, size and cost. A first system implementation and verification is realized by employing the Terasic DE1 board [27]. It contains the EP2C20F484 FPGA module along with appropriate General Purpose Input Output (GPIO) ports and other interesting peripheries such as Synchronous Dynamic RAM (SDRAM), Video Graphic Array (VGA), Universal Asynchronous Receiver/Transmitter (UART), etc. (cf. Figure 6)*.*

The camera is connected with the FEES by employing one of the DE1 GPIO ports. The camera driver of [25] is amended, according to the application requirements, and is employed as a module in the devised system. A specific, cost effective and resource efficient camera driver is designed and implemented in VHDL, instead of employing the commercial camera transceiver Intellectual Properties (IPs) [28]. Later on, it is synthesized on the FPGA. This driver is employed by the FPGA chip to collect images from the camera. The camera configuration can also be changed via this driver [25,29]. Once the system is powered on, the camera driver configures the camera in a desired configuration. The configuration command is located in the system configuration register. After configuration, the camera starts to take images and transmit them to FEES. This data is continuously received by the camera driver and is passed to the data encoder (cf. Figure 6). In [25,29], a comparison is made of the utilization of resources of the devised system.

The output of data encoder is registered on the on-board 8-Mbyte SDRAM [30]. It is done in a real-time fashion via a specifically designed memory controller. This specific implementation adds cost effectiveness and resource utilization efficiency to the system as compared to the employment of commercial SDRAM memory drivers [31]. The devised memory controller manages simultaneous SDRAM read and write access. Moreover, it also passes the data, in a real-time fashion, towards the data decoder module. The decoded data is displayed in a real-time fashion on the VGA monitor.

**Figure 7:**
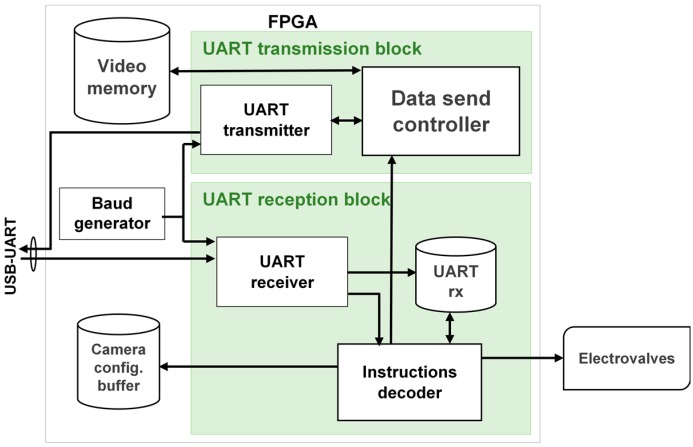
The Universal Asynchronous Receiver/Transmitter (UART) driver block diagram.

The interface between the IPDC unit, runs on the PC, and the FEES, is realized via the USB cable. This interface is controlled by the UART driver. A specific UART driver is realized which results in a cost effective and resources efficient solution compared to the commercially available UART cores [32]. The developed UART driver block diagram is shown in Figure 7.

**Figure 8:**
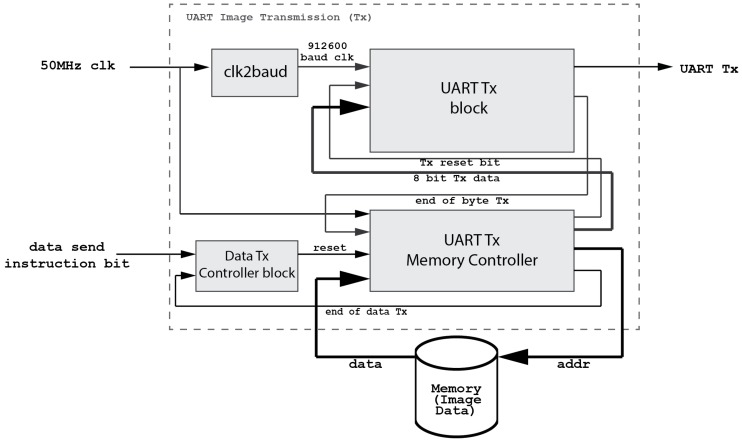
A detailed block diagram of the UART transmitter (Tx).

Figure 7 shows that the devised UART driver is a bidirectional interface. The IPDC sends commands via the USB port and these commands are passed through the onboard USB-UART Bridge to the developed embedded UART module. Similarly, depending on the requirement, the IPDC unit sends an image read command to the FEES. The image data is sent to the embedded UART module, which serializes and packetizes this data following the RS232 protocol (cf. Figure 8). Later on, this data is passed through the onboard UART-USB Bridge. The output of the UART-USB Bridge is passed to the IPDC via the USB cable (cf. Figure 7).

The devised system configures the camera frame rate and resolution according to the application requirements. The targeted application does not require a high frame rate. Therefore, the 912 KBaud rate is employed in the system. However, this implementation supports much higher baud rates as it is successfully tested up to 1 MBauds per second.

### 2.4. The Image Processing, Decision Making and Command (IPDC) Module

**Figure 9:**
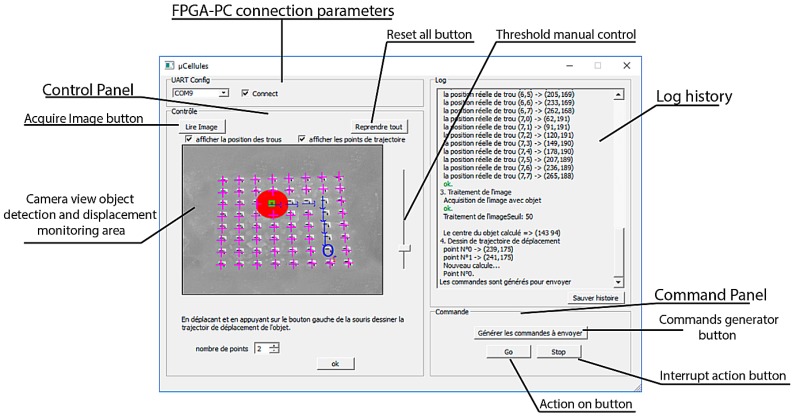
The IPDC Graphical User Interface (GUI) for monitoring and command.

The IPDC is developed in C++. A Graphical User Interface (GUI) is also designed and developed with the QT Creator software [33]. It receives data packets from the FEES and constructs an image from this data (cf. Figure 9).

The IPDC analyzes the received image and compares it with the reference one. The outcomes of this comparison are employed by the IPDC to make decisions. Based on these decisions, the IPDC sends commands to the FEES. Following these commands, the FEES either sends another image to the IPDC unit or it pilots the array of actuators in a desired pattern.

Each instruction set, sent from the IPDC to the FEES, consists of 16 bytes. The first byte contains the main task and the following 15 bytes provide further details. Two different main tasks are assigned to the FEES. The first one is the request for new image data and the second one is the actuators’ piloting request. These instructions are decoded and executed by the Instruction Decoder and Processing module (cf. Figure 6). If the decoded instruction matches the image-send request, then the FEES reads the image from SDRAM and sends it to the IPDC unit via the UART module. In this case, no further instruction details are required. However, for the actuation command, the FEES pilots the valves of the array of actuators one after another. This is done by a sequential execution of the instructions, stored in the instruction buffer. An illustrative example of the actuation command is discussed below.

**Figure 10:**
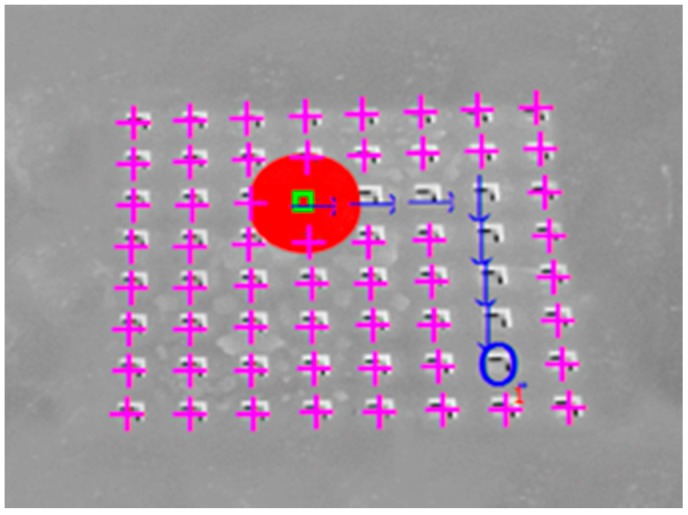
Smart surface Image with an object (red filled circle), solenoid valves holes (“+” signs in violet color), the desired object location (blue circle) and the calculated conveyance trajectory of the object (blue flashes).

The desired object location is marked with a blue circle in Figure 10. Its conveyance trajectory is shown, with the blue arrows, in Figure 10. Each arrow represents a corresponding solenoid valve action command. The process is summarized in Table 1.

The solenoid valve action commands are stored by the FEES, in the instruction buffer, in the sequence to be executed. There is a waiting time between each execution of a solenoid valve action command. It is calculated as a function of the solenoid valves’ response time, the necessary air pressure accumulation time and the considered object weight.

## 3. The Proposed System Functionality

**Figure 11:**
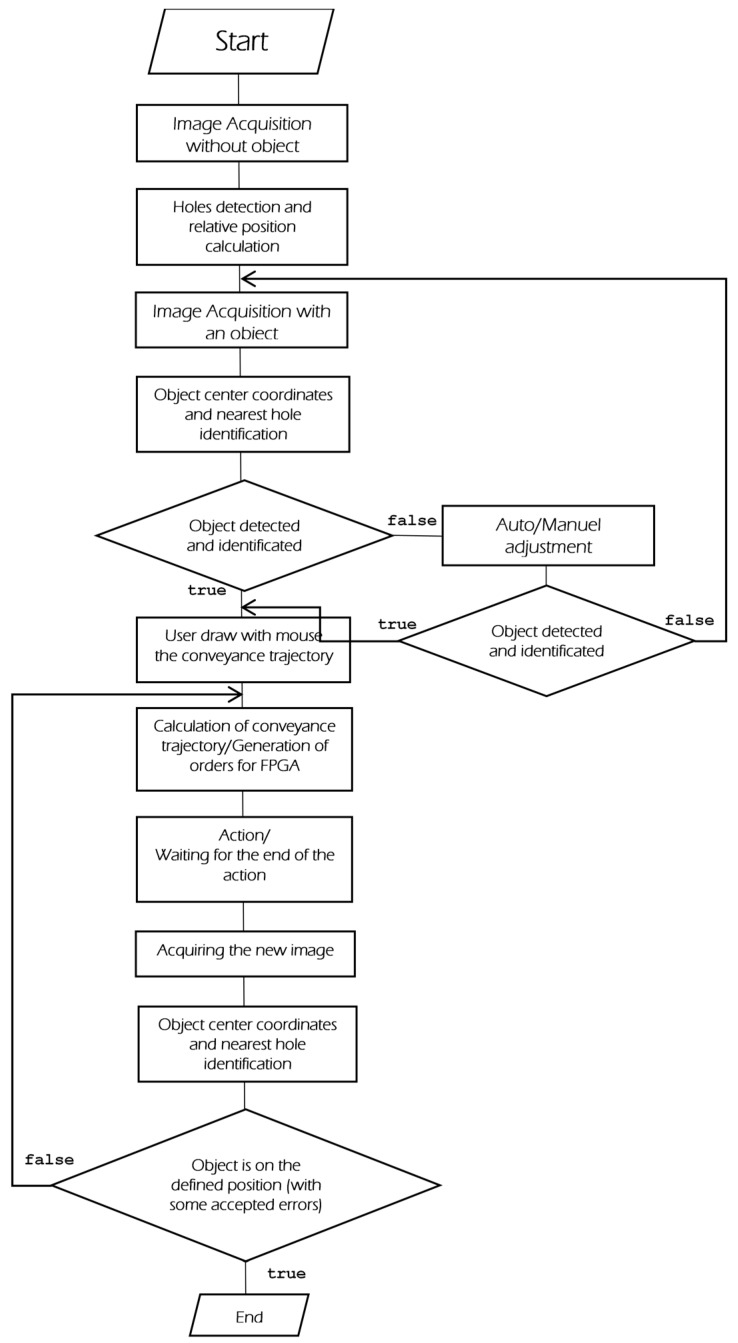
The IPDC unit flowchart.

The proposed system functionality is described with the help of a flow chart, shown in Figure 11. This flow chart consists of the following processes:

- Image acquisition without an object
- Calibration
- Image acquisition with an object present
- Image processing: object position detection
- User-defined conveyance trajectory of the object
- Automated control

A brief description of these processes is given in the following subsections.

### 3.1. Image Acquisition without an Object

**Figure 12:**
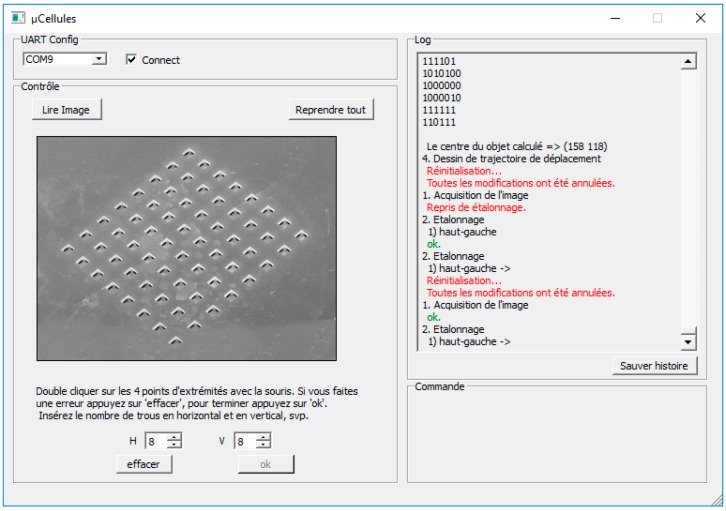
The IPDC unit GUI.

Image acquisition without an object allows the user to calibrate the positions of the solenoid valves’ surface holes (cf. Figure 12). With a left click of the mouse on the GUI, an image request is sent to the FEES. The FEES drives the camera, captures images and sends the data to the IPDC via the UART module.

### 3.2. Calibration

**Figure 13:**
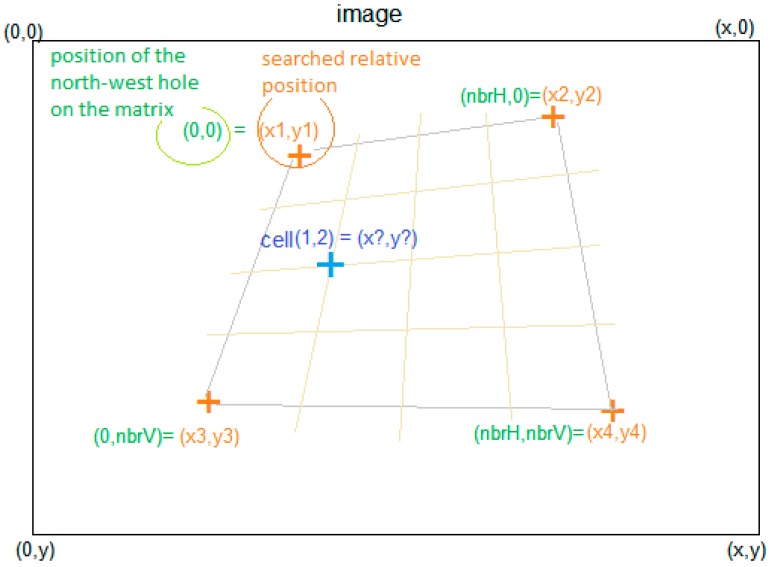
Detection of the positions of smartsurface holes on the captured image.

The calibration process determines the positions of the smart surface holes on the captured image (cf. Figure 13). For this purpose, the user should indicate the positions of the extreme holes. This is done by double clicking respectively on the north-east, north-west, south-east and south-west extreme holes on the smart surface. This process will allow the computation of the smart surface area and the distribution and consecutive distances of different holes on the smart surface. The system is compatible with smart surfaces of different areas and with different numbers of holes. As a function of the extremity holes, marked by the user, the positions of *x*-hole and *y*-hole, relative to the other holes, are determined by employing Equation (1). Here, *i*-hole presents the column number in the matrix and *j*-hole presents the row number in the matrix.

(1) $$x=\;\left(\left(\frac{\left(x_{4}-x_{2}\right)*j}{nbrV-1}+x_{2}\right)-\left(\frac{\left(x_{3}-x_{1}\right)*j}{nbrV-1}+x_{1}\right)\right)*\frac{i}{nbrH-1}+\left(\frac{\left(x_{3}-x_{1}\right)*j}{nbrV-1}+x_{1}\right)\;y=\left(\left(\frac{\left(y_{4}-y_{2}\right)*j}{nbrV-1}+y_{2}\right)-\left(\frac{\left(y_{3}-y_{1}\right)*j}{nbrV-1}+y_{1}\right)\right)*\frac{i}{nbrH-1}+\left(\frac{\left(y_{3}-y_{1}\right)*j}{nbrV-1}+y_{1}\right)$$

In Equation (1), *nbrH* represents the number of horizontal holes (rows) on the smart surface matrix. The *nbrV* represents the number of vertical holes (columns) on the smart surface matrix. The studied smart surface composes of an 8 × 8 matrix of 64 holes. Its surface is 9 mm × 9 mm.

**Figure 14:**
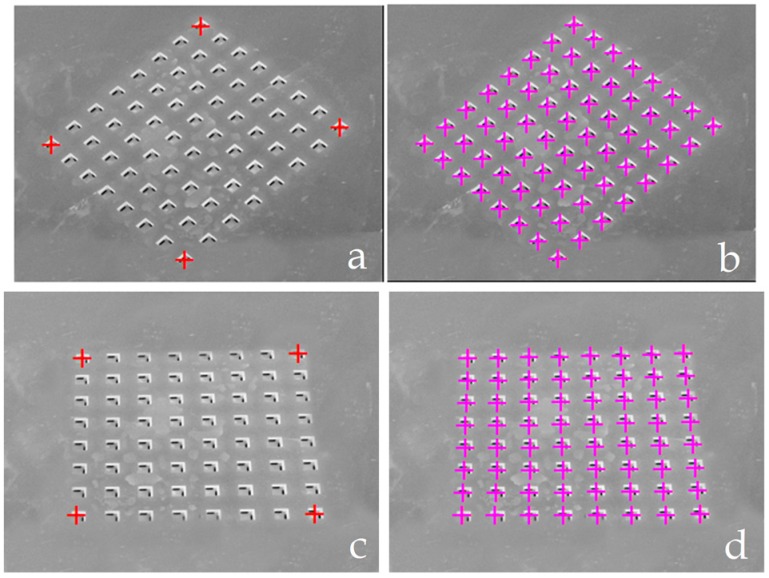
Two different angle views of the smart surface, captured for two different angled embedded camera placements. The extreme holes are presented by red color “+” symbols (**a**,**c**) and others holes are presented by violet color “+” symbols (**b**,**d**).

The smart surface possesses the mirror effect. Therefore, the vertical camera placement should be avoided. The calibration process relaxes the camera placement constraints (cf. Figure 14). However, the camera should be placed at an appropriate angle for tracking the micro-object conveyance. Figure 14 shows two different angle views of the smart surface along with the identification of holes. These views are obtained with two different angled camera placements. However, both camera placements are suitable for proper tracking of micro-object conveyance.

### 3.3. Image Acquisition with an Object

After calibration, the system informs the user, by displaying a message, to place the object on the smart surface and then click on the image button. The FEES follows this command and sends a new smart surface image with an object to the IPDC via the UART module. After receiving the image, the system proceeds to the next step of image processing and the object location determination.

### 3.4. Image Processing

**Figure 15:**
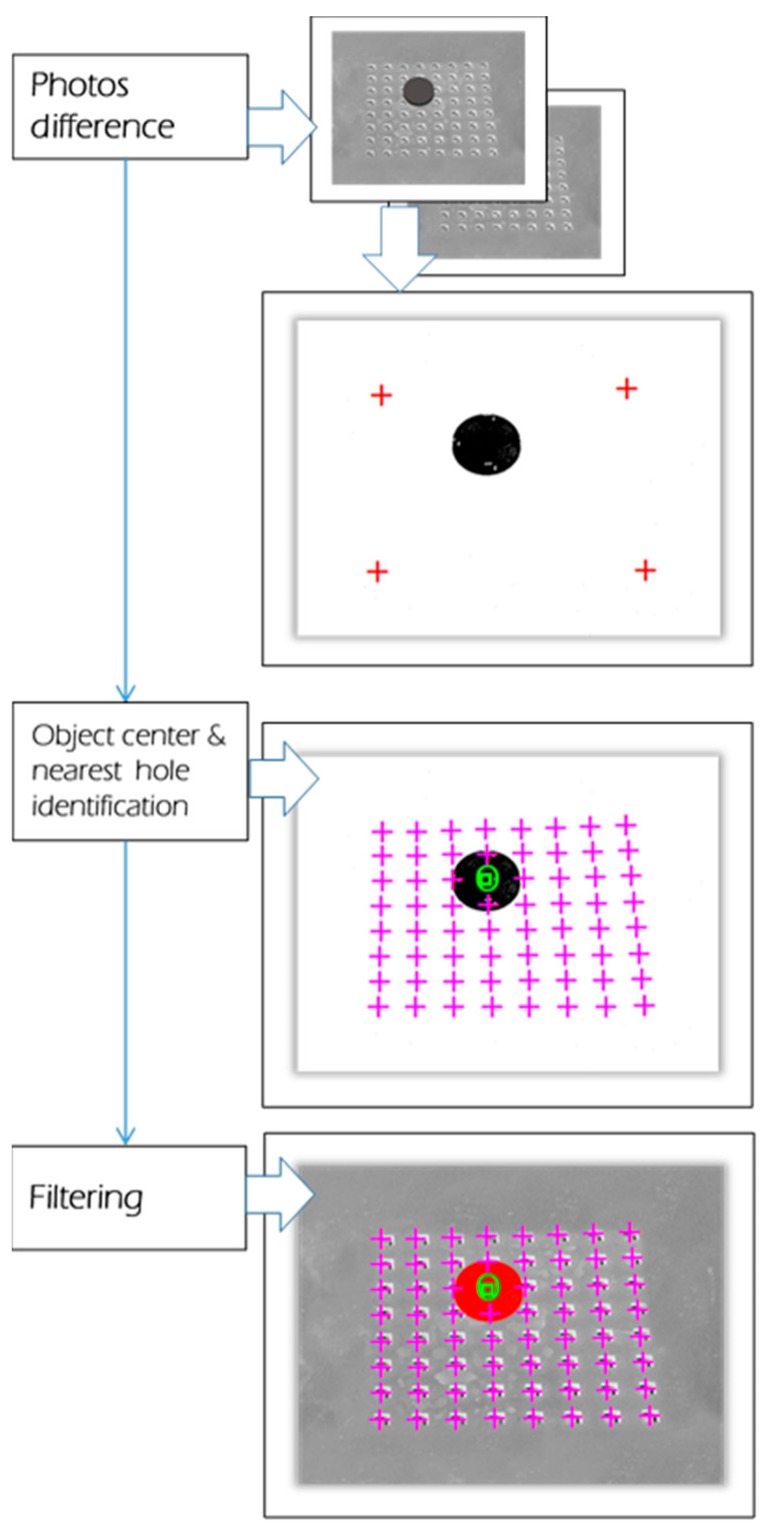
Object detection steps. “□” shows the micro-object center point on the image, “O” shows the nearest identified hole.

An image is a matrix of pixels. Each pixel can present a specific color code. The human brain scans images that are captured by eyes and processes this information in order to extract various features and objects. A similar mechanism is programmed in the proposed machine. Therefore, it can detect different objects on the captured image (cf. Figure 15).

To properly maneuver any object, it is obligatory to know its initial position. In this context, an effective and simple image processing algorithm is used. The different object detection steps are described in Figure 15. The IPDC asks the camera, via the FEES, to capture two pictures of the smart surface: one without and one with an object. The pictures are received in 8-bits grayscale mode. The object detection is realized by employing the BS (Background Subtraction) algorithms [34,35,36,37,38,39,40]. The BS algorithm principle is described with Equation (2).

(2) $$P\left(x,y\right)=\{\begin{array}{c} 1\;\mathrm{if}\;d\left(R\left(x,y\right),O\left(x,y\right)\right)>th)\\ 0\;\mathrm{otherwise} \end{array}$$

In Equation (2), “*P”* is the result, “*d”* is the difference between the reference image “*R”* and object present/moved image “*O”.* The “*th”* is the employed threshold. The “*th”* can be changed by the user via the system GUI (cf. Figure 9).

The BS algorithm working principle is based on the subtraction of each pixel of two pictures and the result is converted into a 1-bit mode. Then, the center of the object is processed by calculating the Root Mean Square (RMS) of the true *x* and *y* pixels’ positions. After the calculation of the object center point, the application identifies the nearest hole to the object center point.

These methods, [34,35,36,37,38,39,40], are simple to implement and are computationally efficient. Therefore, these are good candidates for the embedded applications. However, these algorithms are dependent on the images’ luminosity conditions. However, in our case, it does not matter because the background luminosity conditions are always kept fixed.

In [19], Laurent et al. have chosen to use an open source library “OpenCV” for image processing and object detection. In the case of the proposed system, no special image processing library is employed. The IPDC is based on a specific C++ based implementation. Therefore, it can be easily implemented on an appropriate microprocessor-based embedded platform. Moreover, it can also be easily translated into the VHDL and can be implemented on an appropriate FPGA [37,38,39,40,41].

### 3.5. User-Defined Object Conveyance Trajectory

Using the system’s GUI, the user can indicate the desired location of the object. This position is read by the application and is used to calculate the necessary horizontal and vertical movements. Later on, this information is converted into a sequence of commands, (cf. Table 1), and these commands are sent to the FEES via the UART module. The FEES stocks these commands in the command buffer. When the commands’ reception and storage finishe, the instructions decoder block uses the first byte to determine whether it is a data-send request or an actuation command. In the case of a data-send command, all the following byes are ignored. However, in the case of an actuation command, all the following non-null bytes are executed.

### 3.6. Automated Control

**Figure 16:**
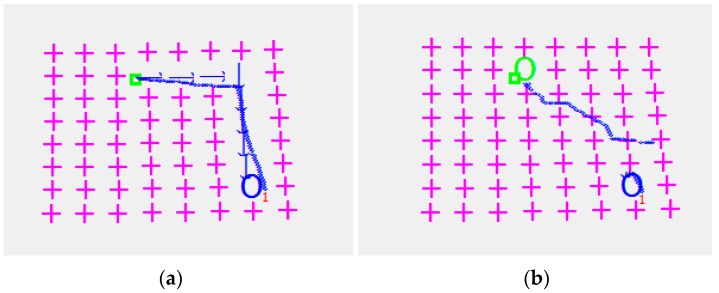
Object displacement algorithm: horizontal and vertical movements (**a**); diagonal movements (**b**).

In the proposed system, each surface hole is piloted by four solenoid valves. Each solenoid valve is independently actuated by an electric signal. These signals are generated by the FEES depending on the IPDC commands (cf. Section 2). These signals control the on/off cycles of electro valves in order to achieve the desired actuation patterns (cf. Figure 16).

## 4. The Experimental Setup and Result

**Figure 17:**
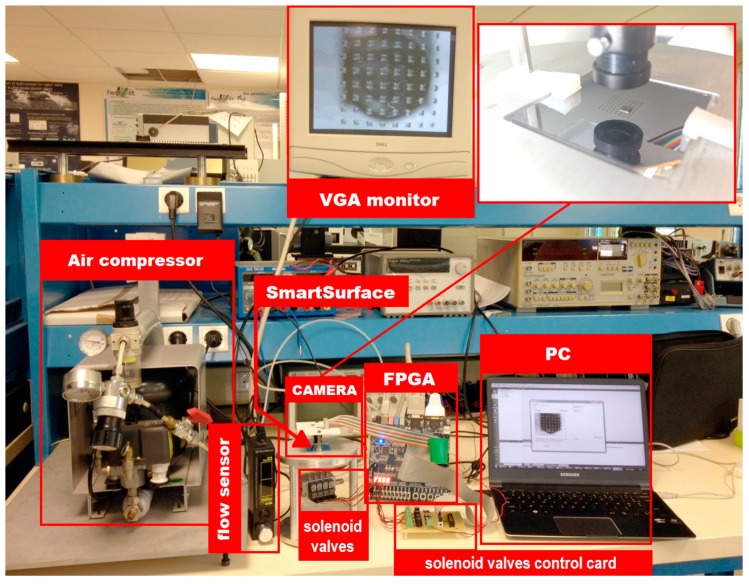
Overview of the experimental setup.

In order to verify the proposed system’s functionality, an experimental setup is devised. It consists of a compressed air supply, four solenoid valves [42], discrete electronics, the 8 × 8 array of actuators [1], a Terasic DE1 card [16], an Omnivision camera OV9655 [43] and a PC to run the IPDC unit. The solenoid valves are connected to the device through fluidic capillaries of 1 mm diameter. Each conveyance path is controlled by one solenoid valve. In this case, a disk of 3 mm diameter and of 100 milligrams weight is employed as the object. Figure 17 shows the complete system setup.

The air-flow source is supplied by compressed air, provided via a pressure control system. The operating pressure is marked as Pin = 20 kPa.

**Figure 18:**
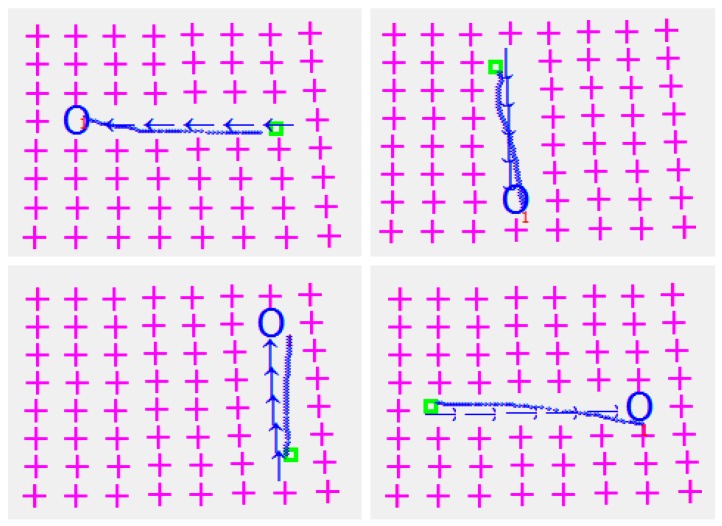
µobject conveyed in four different directions and the deviation errors of its desired and obtain positions. The initial object position is shown by a green color “□” symbol; the blue color arrows show the conveyance trajectory and direction; the blue color symbol “O” indicates the nearest hole from the final object position; the blue color line presents the real trajectory of the object.

The micro-object is conveyed in four different directions and errors between the desired and the obtained object positions are measured. The process is depicted in Figure 18. The arrows in Figure 18 show the steps of the taken decision in order to move the µobject towards the desired location. The blue circles are the µobject desired positions. After analyzing Figure 18, it is clear that there is a deviation between the desired and the obtained final position of the object. The maximum deviation error for the studied system is bounded by 0.4 mm.

The FPGA-based embedded controller manages the micro-object conveyance speed by turning on and off the solenoid valves (cf. Section 3). The maximum opening and closing frequency of the employed valves is 50 Hz [42]. The conveyance algorithm is based on a hole-by-hole movement. Therefore, the maximum achievable conveyance speed in the proposed system is bounded by 20 mm/s. The switching frequency of the system’s solenoid valves is adapted according to the object dimensions (cf. Table 2).

Table 2 summarizes the values of different valve pulse lengths employed for studying the performance of the devised system’s conveyance in the case of different objects. Higher valve pulse length is used for bigger objects because of their weight, and vice versa.

**Figure 19:**
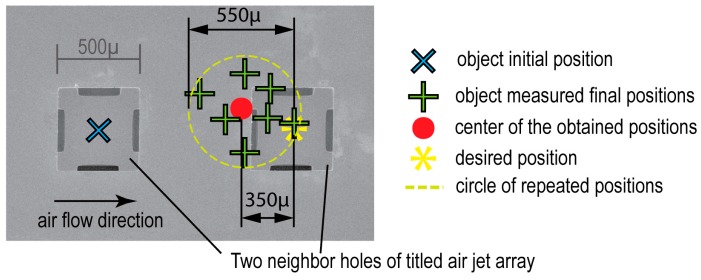
Repeatability and accuracy calculation, based on the difference measurement between the desired and the obtained positions of the µobject.

For the above-mentioned parameters, a repeatability of 550 µm is achieved with a 350 µm accuracy (cf. Figure 19). This is because of the various numbers of holes that remain under the object and participate in its conveyance.

In the case of larger objects, a large number of holes participate in the object conveyance and vice versa. The system is able to maneuver any object with the aforementioned precision and speed, provided that its diameter and weight remain less than or equal to 5.2 mm and 2100 mg respectively. It can also maneuver objects with higher dimensions up to 10 mm in diameter and with a weight of up to 5 g. However, in this case, the conveyance precession and speed could be reduced.

**Figure 20:**
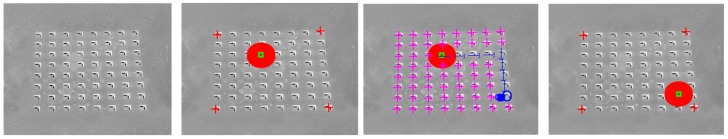
The four main conveyance steps (from left to right): capture of the surface without an object; capture of the surface with an object—image processing and object detection; detection of the desired final position and trajectory calculation; object on the final position.

Figure 20 shows the four captured images, representing the four main steps of the proposed conveyance system respectively.

## 5. Conclusions

In this paper, a fully, FPGA-based, embedded automatic system is devised for systematically piloting the smart surface [1], in order to maneuver the micro-objects. It is a closed-loop system. The feedback, regarding the micro-object’s current location, is provided via the FPGA-based embedded camera to the IPDC (Image Processing, Decision Making and Command) module. The IPDC module compares this micro-object location with the desired one and generates actuation commands. Following these commands, the front-end, FPGA-based, embedded system generates actuation patterns for the smart surface. The same process is repeated in a closed-loop fashion, unless the micro-object achieves the desired position on the smart surface. The IPDC module is based on a specific and profiled C++ based development using QT tool and has no dependencies on the commercial software licenses. The QT tool is compatible with a variety of real-time embedded operating systems such as Linux, IOS, Windows IoT, etc. It ensures the IPDC implementation on an appropriate commercial microprocessor or FPGA-based embedded system. Therefore, the devised solution is independent of a PC-based approach. Moreover, the front-end, embedded system is realized with a specific VHDL-based implementation and it can be implemented on any appropriate FPGA or even an effective ASIC (Application Specific Integrated Circuit) development can be realized. Moreover, a specific, VHDL-based, resource efficient and fully configurable camera driver is employed. The camera frame rate and resolution can be configured via this embedded camera driver with a soft command. This feature is not evidently available with the USB-based standard camera solutions.

The system source codes and the executable files will be provided to other researchers on request.

To conclude, the proposed solution leads to a configurable, smaller sized, cost effective, powerful and resource efficient solution for the targeted application, without any software and hardware license dependencies, compared to the existing solutions.

In the future, the devised system will be used to manipulate the “biomedical micro-objects”. One ongoing project deals with the development of an array of microlenses for miniature imaging systems, manufactured with the same techniques [44]. These lenses require a sophisticated microfabrication process. Therefore, they are quite expensive and their manipulation is quite delicate. This is the reason why we have tested our systems on micro-objects of the same dimensions. In addition, the employment of the proposed system for the conveyance of biosensors, mentioned in [45,46,47], is another prospect.

## Figures and Tables

**Table 1:** Composition of an actuation command.

1 Byte Char	1 Byte Char	1 Byte Char	1 Byte Char	1 Byte Char	1 Byte Char	1 Byte Char	1 Byte Char	1 Byte Char	1 Byte Char	1 Byte Char	1 Byte Char	1 Byte Char	1 Byte Char	1 Byte Char	1 Byte Char
Actuation command	→	→	→	↓	↓	↓	↓	Null	Null	Null	Null	Null	Null	Null	Null

**Table 2:** Solenoid valve pulse length regulation for different object sizes and weights.

Object Diameter (mm)	Object Weight (mg)	Valve Pulse Length (ms)
2.5 3	420 840	50 100
4 5.2	1420 2100	200 350
